# Infected Urachal Sinus in an Adult

**DOI:** 10.7759/cureus.15693

**Published:** 2021-06-16

**Authors:** Hanae Ramdani, Khadija Benelhosni, Nabil MoatassimBillah, Ittimade Nassar

**Affiliations:** 1 Radiology, Ibn Sina University Hospital, Rabat, MAR

**Keywords:** urachus, urachal sinus, infected urachal sinus, congenital anomaly, abdominopelvic computed tomography

## Abstract

Urachal anomalies are infrequent in the adult population and may represent a diagnostic challenge. Few cases of urachal sinuses are reported and are usually asymptomatic unless a complication occurs, most likely infectious. Infected urachal sinus' clinical manifestations are a purulent umbilical discharge, abdominal pain, and a periumbilical mass. We report the case of a late presentation of an infected urachal sinus in a male adult. Clinical and imaging features allowed accurate diagnosis. Antibiotic therapy was followed by the urachal remnant’s surgical excision. The postoperative course was uneventful. Histological examination revealed no signs of malignancy.

## Introduction

Urachal anomalies result from the persistence of urachus, a ductal remnant of the regressed allantois extending from the umbilicus to the apex of the bladder [1]. Four varieties of urachal abnormalities exist patent urachus, umbilical cyst, umbilical sinus, and vesico-urachal diverticulum. Uncommon in adults, they represent a diagnostic challenge [2]. Umbilical discharge/mass, abdominal pain and hematuria are possible clinical presentations. Imaging plays an invaluable role in detection and characterization. Infection and malignancy can occur. We highlight diagnostic and therapeutic features of urachal anomalies through the case report of an infected urachal sinus in a male adult.

## Case presentation

**Figure 1:**
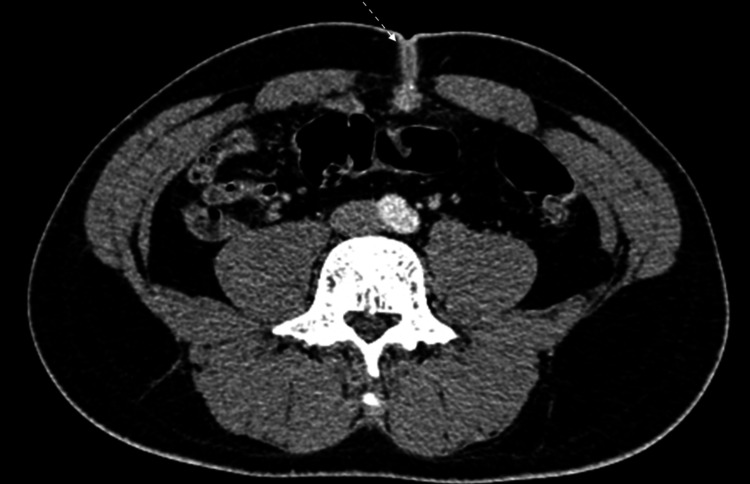
Axial image of a contrast-enhanced abdominal computed tomography showing a thickened walled peripherally enhanced collection (white dotted arrow) extending from the umbilicus towards the pre-peritoneal space.

A 34-year-old male patient presented with moderate mid abdominal pain centered around the umbilicus and purulent umbilical discharge both evolving for seven days. Bladder and bowel habits were not altered. Upon interrogation, the patient reported a similar spontaneously resolving episode in the past six months. Blood work revealed leukocytosis (13600/mm³) and elevated C-reactive protein (56mg/l). The patient was febrile at 101.3°F. Peri-umbilical abdominal regions were tender upon palpation. No lumps were identified. Abdominal ultrasound showed a tubular hypoechoic collection extending from the umbilicus to the infraumbilical area. Contrast-enhanced abdominal CT with umbilical instillation of contrast demonstrated a fusiform blind-ending collection, with peripheral wall enhancement, extending from the umbilicus to the pre-peritoneal space, and measuring 34x11.5mm in size (Figure 1).

**Figure 2:**
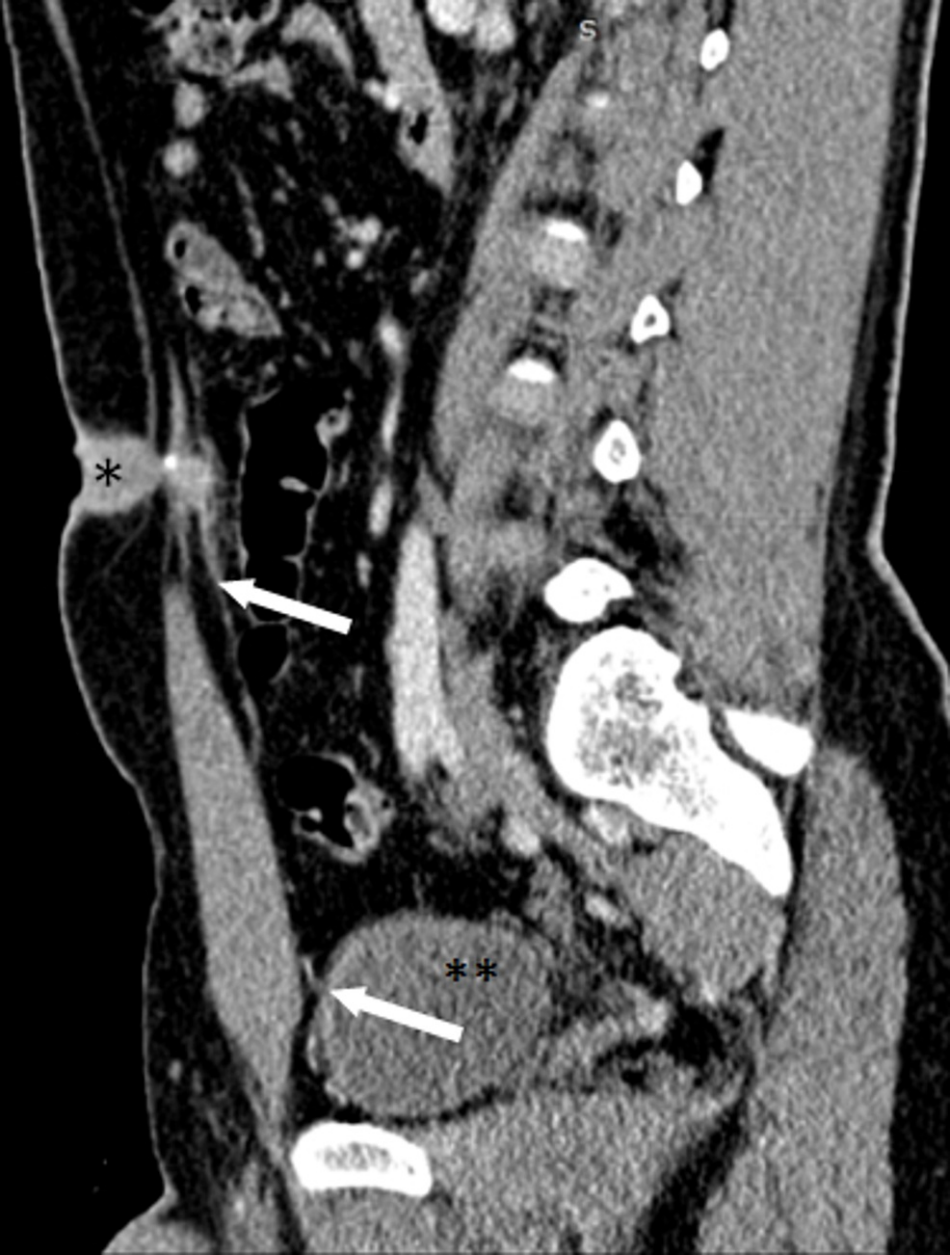
Sagittal reconstruction of a contrast-enhanced abdominal computed tomography showing the tubular collection (*) with a peripheral calcification extending from the umbilicus to the pre-peritoneal space. A linear band (White arrows) connecting the collection’s postero-inferior end to the bladder (**) and corresponding to the medial umbilical ligament is noted.

**Figure 3:**
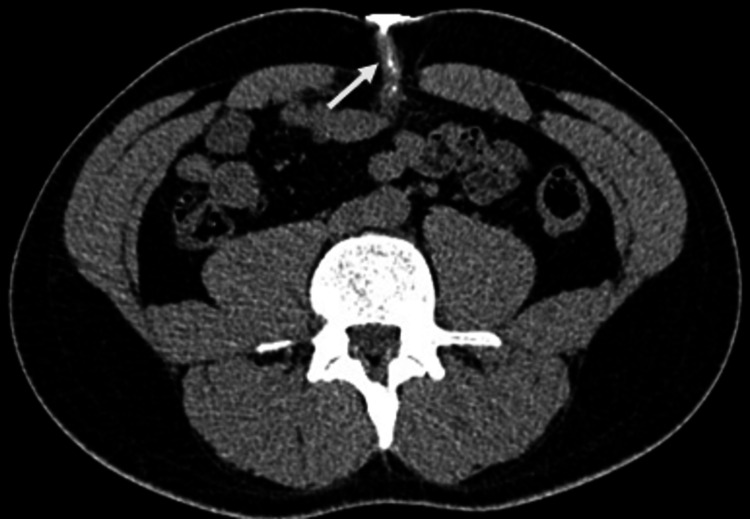
Axial abdominal computed tomography image following iodinated contrast instillation through umbilical opening shows a blind ending partially opacified tubular collection (White arrow) extending posteriorly from the umbilicus towards bladder dome.

**Figure 4:**
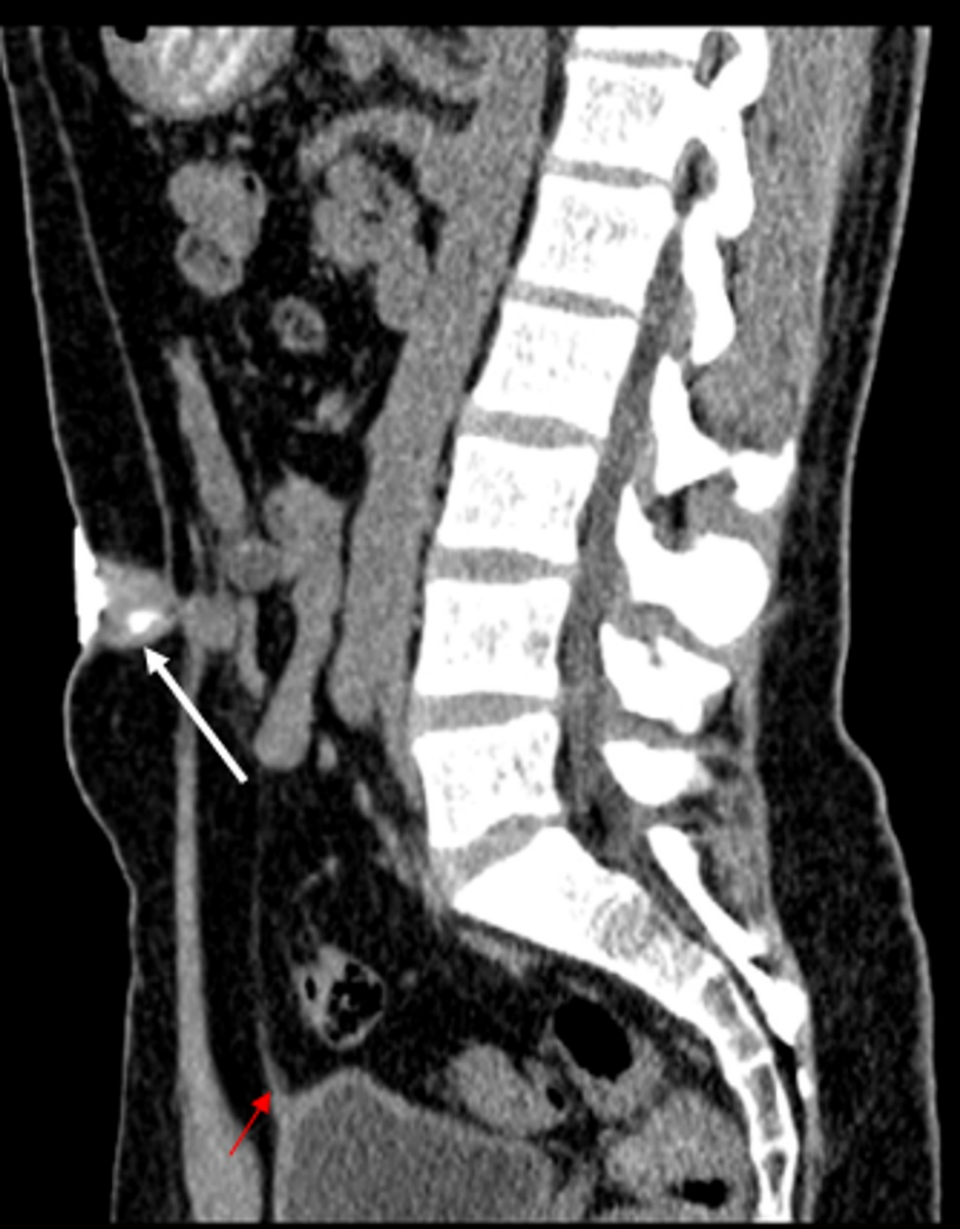
Midsagittal CT scan image following umbilical contrast instillation shows the partially filled tubular collection (White arrow) extending from the umbilicus to the median umbilical ligament (Red arrow). No evidence of communication with the bladder lumen is seen.

A linear band was seen extending from the collection’s posteroinferior end to the bladder’s fundus (Figure 2). Partial opacification of the collection was noted following contrast instillation (Figure 3). No obvious communication with the bladder lumen was noted (Figure 4).

An umbilical discharge swab culture identified Staphylococcus aureus. The patient received an initial dose of antibiotics and was operated on after the infection had cleared. Surgery confirmed the pre-operative diagnosis of a urachal sinus. Sinus excision and umbilectomy were performed. Microscopically, the resected urachal lumen was lined with transitional epithelium and inflammatory cells. No malignancy was detected. Follow-up was uneventful.

The rationale for final diagnosis

Umbilical pus discharge and peri-umbilical pain suggested a urachal anomaly in our patient. Clinically, umbilical discharge aids in diagnosis. Pus discharge may be present with urachal sinus. Urine discharge points to patent urachus, and hematuria suggests vesico-urachal diverticulum. Imaging has a definitive role in classifying the type of urachal anomaly. Our patient's contrast-enhanced CT with umbilical contrast instillation demonstrated a blind-ending tract communicating with the umbilicus, indicative of umbilical sinus. Patent urachus is a free communication between the bladder and umbilicus. Umbilical cyst is the persistence of the central part of the urachal canal presenting as a double-blind-ending cavity. Vesico-urachal diverticulum is a blind-ending tract communicating with the bladder. Fever, pain, pus discharge, and leukocytosis were in favor of a secondary infection, confirmed by the heterogeneous collection aspect on ultrasound and the peripheral collection walls' enhancement on contrast-enhanced computed tomography. No communication between the anomalous tract and the bowel was demonstrated, allowing distinction from the persistence of the omphalomesenteric duct. No palpable mass was present. Radiological features suggestive of malignancy - a mixed solid-cystic appearance, mural nodularity, contiguous structures infiltration, and distant metastasis - were absent.

## Discussion

**Figure 5:**
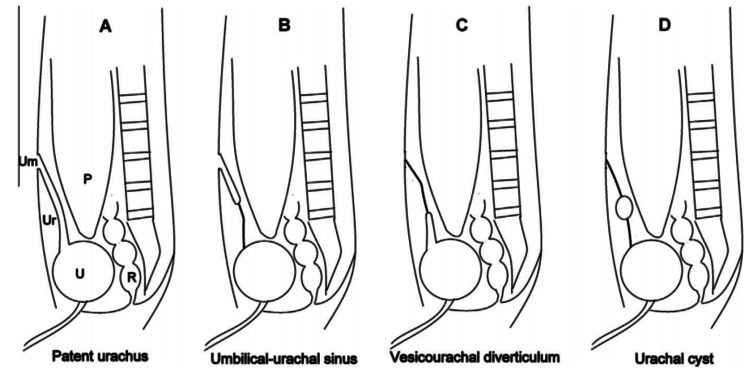
Urachal anomalies graphic representation (A–D). Article under CC BY-NC-ND 4.0 license reused from Kewal Arunkumar Mistry, et al. (2015) [3]. Um-umbilicus, Ur-urachus, U-urinary bladder, R-rectum, P-peritoneal cavity

The urachus is a ductal remnant extending from the bladder's anterior end to the umbilicus. It originates from the allantois and the cloaca's involution. It is normally obliterated in utero or early childhood and becomes the medial umbilical ligament [1]. Its persistence gives rise to a variety of urachal anomalies: Patent urachus (50%) : a free vesico-umbilical communication, Umbilical cyst (30%) : a remaining double blind ending cavity of the urachal canal, Umbilical sinus (15%) : a blind-ending tract communicating with the umbilicus, Vesico-urachal diverticulum (3-5%) : an obliterated tract communicating with the bladder (Figure 5).

Urachal abnormalities' incidence in children is 1 in 5000 and male to female ratio 3:1. Occurrence in adults is uncommon (2 per 100.000 hospitalizations) and constitutes a diagnostic challenge [2]. Umbilical discharge/mass, abdominal pain, and hematuria are possible clinical presentations. Umbilical discharge aids in diagnosis. Pus discharge points towards urachal sinus. Urine discharge indicates patent urachus, and hematuria suggests vesico-urachal diverticulum.

Imaging plays an invaluable role in urachal anomalies detection and characterization. Ultrasound is the modality of choice for initial screening. Other recommended imaging modalities are sinography, CT and Magnetic resonance imaging [4]. In the setting of an umbilical-urachal sinus: US, CT and MRI demonstrate a tubular blind-ending tract extending the umbilical end. Sinography confirms the lack of communication with the bladder. A bladder communication is diagnostic of a patent urachus. Urachal cysts appear as fluid-filled structures along the urachus theoretic course. Vesico-urachal diverticula extend the anterior bladder dome and do not communicate with the umbilicus.

In cases of infection, the US demonstrates a urachal remnant with complex echogenicity. CT shows heterogeneous attenuation with generally augmented contrast enhancement. MRI can assist in infection's extent determination (bladder and contiguous structures' implication). Exceptionally, severe presentations occur: complex fistulae and abscesses form with the risk of intraperitoneal spillage, peritonitis and sepsis.

Urachal neoplasms are rare. Benign urachal tumors (Adenomas, Cystadenomas, Fibromas, Fibromyomas, Fibroadenomas, and Hamartomas) mimic malignancy. The final diagnosis is anatomopathological. On cystography, extrinsic compression or a bladder's dome filling defect appears. Ultrasound shows a midline heterogeneous soft-tissue mass with calcifications. CT and MRI evaluate tumor's extension locally, assess lymph nodes involvement and distant metastases in case of suspected malignancies. CT shows a midline cystic, solid or mixed mass contiguous to the bladder's dome with a low attenuation mucin component and calcifications. Of note, a midline soft-tissue mass along the urachus course with calcifications is pathognomonic of urachal adenocarcinoma. MRI reveals a heterogeneous midline mass, best visualized on sagittal images, with focal zones of high signal intensity on T2-weighted images corresponding to mucin and solid components, isointense to soft-tissue on T1-weighted images, enhancing following Gadolinium administration [5].

When not detected rapidly and treated promptly, urachal pathology can cause significant morbidity. Infection is the most common complication in adults as in the pediatric population. Malignancy may develop as well in the adult population. Other complications include umbilical granulomas and lasting urinary infections. Calcifications may arise due to urinary stasis and cyst wall's glandular epithelium calcification. They most commonly involve urachal cysts and vesicourachal diverticula. A progressively enlarging urachal cyst can rupture, obstruct intestines, or fistulize into the adjacent bowel [5].

Congenital urachal anomalies' management is controversial. Prophylactic surgical excision of asymptomatic urachal remnants' benefits is debated. Infants under one year of age are treated surgically in case of repetitive symptoms or failure of spontaneous resolution. In older symptomatic patients, the recommended approach is the urachal remnant's complete laparoscopic or open surgery excision, although some authors advocate a conservative strategy with follow-up irrespective of age. Anomalies extending to the bladder's dome require segmental vesical resection [6]. 

## Conclusions

Infected urachal sinus is uncommon in adults. Diagnosis is challenging requiring a high index of clinical suspicion. A peri-umbilical mass, umbilical discharge, and sepsis are suggestive. Ultrasound and computed tomography scans' diagnostic role is invaluable. Antibiotherapy followed by surgical intervention prevent recurrence and malignant transformation risk.
